# Will Organoids Fill the Gap towards Functional Precision Medicine?

**DOI:** 10.3390/jpm12111939

**Published:** 2022-11-21

**Authors:** Federica Papaccio, Manuel Cabeza-Segura, Blanca Garcia-Micò, Noelia Tarazona, Desamparados Roda, Josefa Castillo, Andres Cervantes

**Affiliations:** 1 Department of Medicine, Surgery and Dentistry “Scuola Medica Salernitana”, University of Salerno, Via S. Allende, 84081 Baronissi, Italy; 2 Biomedical Research Institute INCLIVA, Hospital Clínico Universitario, Department Medical Oncology, University of Valencia, 46010 Valencia, Spain; 3 Centro de Investigacion Biomedica en Red (CIBERONC), Instituto de Salud Carlos III, 28029 Madrid, Spain; 4 Department of Biochemistry and Molecular Biology, University of Valencia, 46010 Valencia, Spain

**Keywords:** precision medicine, functional precision medicine, organoids, solid tumors

## Abstract

Precision medicine approaches for solid tumors are mainly based on genomics. Its employment in clinical trials has led to somewhat underwhelming results, except for single responses. Moreover, several factors can influence the response, such as gene and protein expression, the coexistence of different genomic alterations or post-transcriptional/translational modifications, the impact of tumor microenvironment, etc., therefore making it insufficient to employ a genomics-only approach to predict response. Recently, the implementation of patient-derived organoids has shed light on the possibility to use them to predict patient response to drug treatment. This could offer for the first time the possibility to move precision medicine to a functional environment.

## 1. Introduction

In the last decades, huge efforts have been made on the molecular characterization of solid tumors, condensed in The Cancer Genome Atlas project. Since the introduction of the first molecularly targeted agent trastuzumab, an increasing number of drugs have been approved based on the presence of a particular molecular alteration, mostly represented by hotspot mutations or copy number variations in driver genes, opening the field for precision medicine in oncology. According to the European Society of Medical Oncology (ESMO), precision medicine can be defined as “a healthcare approach with the primary aim of identifying which interventions are likely to be of most benefit to which patients based upon the features of the individual and their disease. In cancer, the term usually refers to the use of therapeutics that are expected to confer benefit to a subset of patients whose cancer displays specific molecular or cellular features” [1]; in summary “the right drug, to the right patient, at the right time”.

Recently, the introduction of patient-derived organoids (PDOs) (stable primary cultures derived directly from the patient) showed the possibility of using them to predict in vitro the response that should be observed in the patient. This could represent a way to test the effectiveness of a precision medicine approach before administering it to the patient, maximizing clinical benefit.

## 2. What Has the Clinical Impact of an Approach Based on Precision Medicine Been So Far?

Thanks to the implementation of modern next-generation sequencing (NGS) technologies and, in general, to improved knowledge of the molecular basis of neoplasms, over the past 20 years we have seen a disruptive and growing approval of molecularly targeted drugs. Alongside well-known molecular targets, new ones are constantly being identified, so much so that today there are more than 30 promising genomic targets. Such a list is just a first iteration of an ongoing process, and the number of targets is expected to continue to expand at a rapid pace. However, the final number of genes that could be drug targeted is difficult to predict. The cumulative work establishing these targets has already significantly increased the fraction of patients who are now believed to carry at least one potentially targetable genomic alteration. The number of tumors harboring at least one targetable alteration is constantly increasing, as is the number of potentially druggable alterations [2], therefore increasing the complexity of molecularly guided treatment approach in solid tumors.

However, what is the clinical impact of an approach based on precision medicine? On closer inspection, the clinical studies conducted so far have proved to be rather disappointing, not being able to reach the set endpoints, either due to difficulties related to the often heavily pretreated study population, or to difficulties related to the execution times of genomic analyzes, etc. For instance, the phase 2 SHIVA trial [3] randomized patients to receive a matched molecularly targeted agent or treatment according to physician’s choice. It did not show a benefit in terms of progression-free survival (PFS). Moreover, the MOSCATO-01 trial highlighted the fact that often a very small percentage of patients effectively benefit from this approach [4]. Similar findings were also shown when liquid biopsy was used to detect molecular alterations in the TARGET study [5]. Disappointing results in terms of objective response rate and feasibility were shown by the NCI-MPACT trial [6].

Albeit substantially negative, from these studies a strong propulsive thrust emerges, evidenced by the exceptional responses seen in individual cases and based precisely on precision medicine approaches. Indeed, encouraging data come from the more recent MAST study [7], where patients in the molecularly guided treatment arm had a higher PFS, and the percentage of patients able to receive further treatments was double in the experimental arm.

Collectively, the clinical impact of a precision medicine approach has not reached the level that was expected. One the most important issues is represented by the fact that most personalized oncology approaches rely on genomics, although it is of crucial relevance to consider that genomics does not automatically predict the response to a given treatment. Indeed, multiple factors can influence the response in the individual patient: the coexistence of other molecular alterations, gene expression, protein modifications, the tumor microenvironment, microbiota, the activation of alternative molecular pathways, and other less explored mechanisms. These factors contribute to interpatient heterogeneity [7] in response, even when the same genomic alteration is shared.

This heterogeneity in response is certainly the epiphenomenon of the various mechanisms that contribute to the heterogeneity that is not only inter- but also intra-tumoral. Indeed, this year Hanahan added phenotypic plasticity, epigenetic reprogramming, senescence, and the role of the microbiota to the fundamental Hallmarks of Cancer work [8].

## 3. The Need to Advance Functional Precision Medicine

In this context, precision medicine approaches should move from a static environment to a dynamic one, leading to the so-called functional precision medicine [9]. This is focused on the possibility to treat patient-derived cancer cells with a given drug, which should be administered when an in vitro response is observed. This is why Letai proposes a new approach to precision medicine, which should take place through the integration of several techniques, where drugs are selected on the basis of genomic and functional tests performed directly on the tumor sample of the individual patient [10]. Therefore, functional precision medicine can be defined as a strategy whereby live tumor cells of affected individuals are directly perturbed with drugs in order to provide personalized information that can be immediately translated to guide therapy [9]. For this reason, it is essential to choose the most appropriate model to use.

Indeed, for many years this kind of approach has been technically impossible, as primary cell lines typically were cultured only for a few passages, therefore impeding drug screening approaches. Recently, this situation has been completely revolutionized by the development of a new primary culture technique, represented by the so-called organoids.

Organoids are 3D primary cultures that can be derived from both healthy and pathological patient tissue and can be grown over a long period of time, while maintaining genomic stability. They are in a more physiologically relevant condition than cell lines; cell-cell and cell-matrix interactions are preserved; they can be expanded, so they are perfectly suitable for drug screening; they can be used for genome editing; and they are perfect models for studying development and in particular human diseases. Therefore, they quickly became key models for translational research as well.

Organoids derived from human tissues have the advantage of being representative of human physiology, unlike animal models, which are only similar to it, they can be established in a quicker (compared to, for instance, patient-derived xenografts, PDXs) and simpler manner, they can be used for large-scale genetic screening as well as for drug screening, they can be genetically manipulated according to the most modern techniques, and since they are obtained by single individuals, they allow biological phenomena to be studied in a personalized way.

The development of organoids started thanks to identification of the Lgr5+ adult stem cells population within the intestinal crypt [11], which led to the development of the growth factor recipe for human intestinal organoids [12]. This opened the way to the generation of organoids from many tissues, through slight modification of organoid media composition, leading to the development of so-called “living biobanks” of organoids from many healthy and diseased tissues.

Human organoids are increasing knowledge in the field of oncology, thanks to the isolation of organoids derived from tumor tissues. Recent studies describe the genomic and morphological characterization of tumor organoids from biopsies of colon [13,14,15,16,17], brain [18], prostate [19], pancreas [20,21], liver [22], breast [23], stomach [24], esophagus [25], endometrium [26], lung [27,28], and the head and neck [29]. These studies have shown that organoids maintain the same characteristics of the original tissue in culture; for instance, showing that PDOs maintain the same immunohistochemical markers of original tissue, therefore showing that the cell population is similar. In particular, while all the studies reported on genomic profile, showing that PDOs replicate both the mutation and copy number profile, only a few studies have reported on the transcriptomic similarities [25] and none of them have reported on proteomics. More relevant for precision medicine, many studies showed that PDOs can replicate the response to pharmacological treatments, with the study by Vlachogiannis et al. reporting an outstanding 100% sensitivity, 93% specificity, 88% positive predictive value, and 100% negative predictive value for PDOs in predicting response in patients [17].

## 4. The Impact of Organoids for Functional Precision Medicine

The implementation of PDOs could represent the key towards functional precision medicine, allowing in the future non-responding patients to be spared side effects in the case of drugs that are already in the clinic or at an advanced development stage, but also allowing for the functional validation of emerging genomic and non-genomic biomarkers and the potential to screen new active compounds in models that are a faithful reproduction of the tumor, therefore potentially shortening the path of drug development.

Clearly, this technology is in its infancy, although it is extremely promising. The development of organoids from adult tissues is limited by the accessibility of the tissue of interest and by the knowledge of the culture conditions of that particular tissue. Nevertheless, there are some disadvantages, which can be interpreted as challenges for improving the technology: the absence of the microenvironment, particularly in organoids derived from adult stem cells, and the absence of standardized protocols and quality controls for which results may vary between research groups. Although cheaper than mouse models, they are nevertheless much more expensive than cell lines. Furthermore, studies at the organ level are complex.

Perhaps what is most important for the implementation of organoids as a functional precision medicine tool, more than a “simple” genomic similarity, is the proof that they “behave” just like the patient’s tumor, specifically their capacity to replicate and predict treatment outcome. A good starting point and example is represented by what has been done for colorectal cancer (CRC). In a 2019 paper, a library of PDOs derived from metastatic CRC patients has been shown to predict response to irinotecan-based chemotherapy and not to 5-fluorouracil plus oxaliplatin [30]. Therefore, these authors suggested that PDOs should be used to select those patients who could benefit from irinotecan from those who would only experience toxicity. In a more recent work, authors aimed at studying heterogeneity in the metastatic setting from a functional point of view in order to test if different PDOs lines derived from different metastatic sites of the same patient would respond differently to drug treatment. This is one of the key clinical questions, as mixed responses are often observed in the clinic. Surprisingly, intra-patient inter-metastatic heterogeneity in drug response was not observed [16].

However, all these studies (and those conducted for other tumor types) were observational. The first interventional clinical study was the single-center, single-arm SENSOR study [31] with the aim of evaluating the feasibility of PDOs to assign patients to treatment with off-label or experimental agents. The primary endpoint was an objective response rate greater than or equal to 20%. Patients underwent a culture biopsy before starting their last standard of care course. The organoids were exposed to a panel of eight drugs targeting main driver pathways, and patients were treated after progression on standard treatment after having identified a clear signal of antitumor activity in vitro. Sixty-one patients were included, and thirty-one organoids were generated from fifty-four eligible patients. Twenty-five cultures were screened for drugs and nineteen organoids showed substantial responses to one or more drugs. Three patients were treated with the mTOR inhibitor vistusertib and three were treated with the pan-Akt inhibitor capivasertib. Despite the drug sensitivity shown by organoids, patients did not demonstrate objective clinical responses to the recommended treatment. The authors concluded that this strategy is not feasible. However, of the 19 patients for whom a significant activity was shown for at least one drug in the PDO, only six started a treatment, while most of the remaining ones dropped out of the study due to clinical deterioration. Maybe the disease setting (patients without curative treatment options, heavily pretreated) is something that per se limited the accessibility of the treatment, together with the 10 weeks necessary to perform all the procedures, from PDOs generation to drug screening. What we can learn from this experience is the necessity to invest in improving the method: for instance, accelerating the process of developing organoids and maximize the performance of drug screening.

Another relevant setting for which organoids could give a significant contribution is represented by locally advanced rectal cancer (LARC), where treatment choice relies on clinical and radiological features [32,33] without biomarkers of response available, and where organ-preserving strategies are under closer evaluation. In this context, the possibility to predict patient response could be crucial to spare unnecessary side effects, plan a better treatment strategy, and to better select patients for conservative strategies.

For instance, Ganesh et al. [34] established a biobank of pre- and post-treatment rectal cancer organoids with a success rate of 77%. Forty-nine lines were generated using tissue obtained with biopsy routinely used in clinical care. Of these, 22 were derived from treatment-naïve patients. Organoids were treated with both standard chemotherapy and radiation therapy, with a good correlation with patient’s response.

In a following larger study by Yao et al. [35], organoids were generated from LARC patients before any treatment, establishing a total of 92 lines from 112 patients. Eighty lines were treated with radiation, 5-fluorouracil, and irinotecan. Interestingly, PDOs were able to reproduce clinical response, and patients achieved a good response when their organoids were sensitive to at least one agent. These data are encouraging, although no clinical data exist for single agent use nor for 5-fluorouracil and irinotecan combinations.

Clearly, future studies should better replicate the treatment strategies that are part of the standard treatment for LARC.

## 5. Future Perspectives

Of importance, there is a great need to standardize the procedures to develop and maintain organoids as well as to subject them to drug screening, otherwise results will never be comparable. To achieve this, a great effort is needed from both academic and pharma industries, which can be attracted by the great potential of this platform even for drug development. The value of PDOs for drug development has been recently proven in a paradigmatic study where CRC PDOs were employed for large-scale screening compounds of bispecific antibodies targeting Wnt and RTK and resulting in the development of an effective compound, MCLA-158, capable of inducing EGFR degradation in Lgr5+ cancer stem cells and inhibiting the growth of KRAS mutant CRC [36]. These unprecedented results should encourage others to apply this approach to drug development in a systematic way.

Nevertheless, a lot of questions remain unanswered. First, uniform protocols are needed to homogenize and standardize culture conditions and success rates, which are quite different among different research groups, and to select the best conditions for drug screening and drug screening interpretation. A key issue is represented by the way to correlate in vitro with in vivo response. Current models are deprived of stromal and immune microenvironment; therefore, its possible effect on drug response is lost. Many efforts are undergoing to develop co-cultures that will allow, for instance, the incorporation of immunotherapy agents into drug screening platforms.

Clearly, the technology is still in its infancy, and many additional studies are needed to develop its use in functional precision medicine. The clinical trials of the future could include, in addition to an extensive molecular characterization, the analysis of PDO response to guide the allocation of patients to different treatment arms.

## Data Availability

Not applicable.
